# (Carbonyl-1κ*C*)bis­[2,3(η^5^)-cyclo­penta­dien­yl][μ_3_-(*S*-methyl trithio­carbonato)methylidyne-1:2:3κ^4^ *C*,*S*′′:*C*:*C*](triphenyl­phosphine-1κ*P*)(μ_3_-sulfido-1:2:3κ^3^ *S*)dicobalt(II)iron(II) trifluoro­methane­sulfonate

**DOI:** 10.1107/S1600536808008970

**Published:** 2008-04-10

**Authors:** Anthony R. Manning, C. John McAdam, Anthony J. Palmer, Jim Simpson

**Affiliations:** a Department of Chemistry, University College Dublin, Belfield, Dublin 4, Ireland; b Department of Chemistry, University of Otago, PO Box 56, Dunedin, New Zealand

## Abstract

The asymmetric unit of the title compound, [FeCo_2_(C_5_H_5_)_2_(C_3_H_3_S_3_)S(C_18_H_15_P)(CO)]CF_3_SO_3_, consists of a triangular irondicobalt cluster cation and a trifluoro­methane­sulfonate anion. In the cation, the FeCo_2_ triangle is symmetrically capped on one face by an S atom and on the other by a C atom linked to a methyl trithio­carbonate residue that bridges the Fe—C bond. Each Co atom carries a cyclo­penta­dienyl ligand while the Fe atom coordinates to one carbonyl and one triphenyl­phosphine ligand. In the crystal structure, the cation is linked to the anion by a number of weak non-classical C—H⋯O and C—H⋯F hydrogen bonds and weak S⋯O (3.317 Å) and S⋯F (3.198 Å) inter­actions. The structure is further stabilized by additional inter­molecular C—H⋯O, C—H⋯F and O⋯O (2.942 Å) contacts, together with an unusual S⋯π(Cp) inter­action (S⋯centroid distance = 3.385 Å), generating an extended network.

## Related literature

For the preparation of the title compound, see: Manning *et al.* (2003 ▶). For reference structural data, see: Allen *et al.* (1987 ▶, 2002 ▶). For related sulfur- and carbon-capped triangular FeCo_2_ structures, see: Manning, O’Dwyer *et al*, (1995 ▶, 1998 ▶, 1999 ▶); Manning, Palmer *et al.* (1998 ▶). For related literature, see: Ringer *et al.* (2007 ▶).
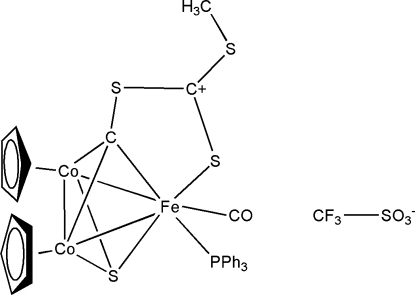


## Experimental

### 

#### Crystal data

[FeCo_2_(C_5_H_5_)_2_(C_3_H_3_S_3_)S(C_18_H_15_P)(CO)]CF_3_SO_3_
*M* *_r_* = 910.53
Monoclinic, 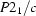

*a* = 11.0403 (6) Å
*b* = 29.2183 (14) Å
*c* = 10.9040 (5) Å
β = 100.664 (3)°
*V* = 3456.7 (3) Å^3^
*Z* = 4
Mo *K*α radiation
μ = 1.77 mm^−1^
*T* = 91 (2) K
0.18 × 0.06 × 0.06 mm

#### Data collection

Bruker APEXII CCD area-detector diffractometer
Absorption correction: multi-scan (*SADABS*; Bruker, 2006 ▶) *T*_min_ = 0.717, *T*_max_ = 0.899
36119 measured reflections
5502 independent reflections
4297 reflections with *I* > 2σ(*I*)
*R*_int_ = 0.081

#### Refinement

*R*[*F* ^2^ > 2σ(*F* ^2^)] = 0.031
*wR*(*F* ^2^) = 0.067
*S* = 1.03
5502 reflections
443 parameters
H-atom parameters constrained
Δρ_max_ = 0.38 e Å^−3^
Δρ_min_ = −0.35 e Å^−3^

### 

Data collection: *APEX2* (Bruker 2006 ▶); cell refinement: *APEX2* and *SAINT* (Bruker 2006 ▶); data reduction: *SAINT*; program(s) used to solve structure: *SHELXS97* (Sheldrick, 2008 ▶) and *TITAN2000* (Hunter & Simpson, 1999 ▶); program(s) used to refine structure: *SHELXL97* (Sheldrick, 2008 ▶) and *TITAN2000*; molecular graphics: *ORTEP-3* (Farrugia, 1997 ▶) and *Mercury* (Macrae *et al.*, 2006 ▶); software used to prepare material for publication: *SHELXL97*, *enCIFer* (Allen *et al.*, 2004 ▶) and *PLATON* (Spek, 2003 ▶).

## Supplementary Material

Crystal structure: contains datablocks global, I. DOI: 10.1107/S1600536808008970/hb2713sup1.cif

Structure factors: contains datablocks I. DOI: 10.1107/S1600536808008970/hb2713Isup2.hkl

Additional supplementary materials:  crystallographic information; 3D view; checkCIF report

## Figures and Tables

**Table:** 

Co2—S1	2.1275 (9)
Co1—S1	2.1564 (9)
Fe1—S1	2.1836 (9)
Fe1—C1	1.891 (3)
Fe1—Co1	2.5035 (6)
Fe1—Co2	2.6149 (6)
Co1—C1	1.867 (3)
Co1—Co2	2.4153 (6)
Co2—C1	1.880 (3)

**Table:** 

Co2—S1—Co1	68.64 (3)
Co2—S1—Fe1	74.67 (3)
Co1—S1—Fe1	70.45 (3)
Co1—C1—Co2	80.27 (13)
Co1—C1—Fe1	83.54 (13)
Co2—C1—Fe1	87.81 (13)

**Table 2:** Hydrogen-bond geometry (Å, °)

*D*—H⋯*A*	*D*—H	H⋯*A*	*D*⋯*A*	*D*—H⋯*A*
C3—H3*A*⋯O2	0.98	2.56	3.477 (4)	156
C3—H3*C*⋯F3	0.98	2.62	3.297 (4)	127
C11—H11⋯O3	0.95	2.41	3.293 (4)	154
C21—H21⋯O2	0.95	2.64	3.588 (4)	174
C21—H21⋯O3	0.95	2.64	3.235 (4)	121
C13—H13⋯O1^i^	0.95	2.42	3.288 (4)	152
C14—H14⋯F1^i^	0.95	2.56	3.248 (4)	129
C35—H35⋯O1^ii^	0.95	2.53	3.283 (4)	136
C24—H24⋯O2^iii^	0.95	2.49	3.298 (4)	143

Symmetry codes: (i) 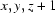; (ii) 
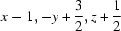
; (iii) 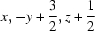.

**Fig. 1.**
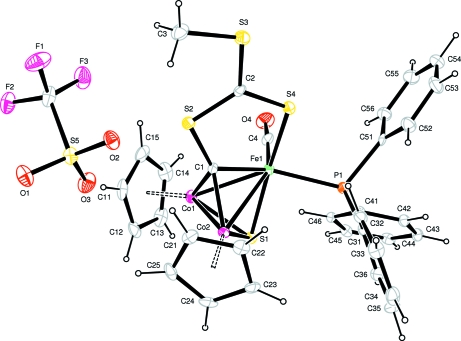
The asymmetric unit of (I), with 50% probability displacement ellipsoids for non-H atoms.

**Fig. 2.**
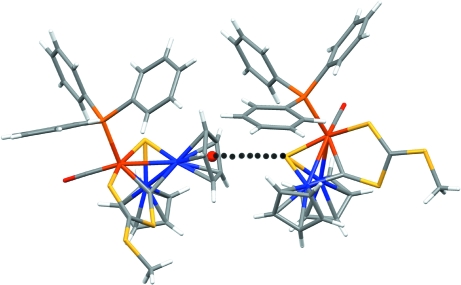
The unusual S···π(Cp) interaction (dotted line) in (I). The red circle represents the centroid of the C21···C25 cyclopentadiene ring.

**Fig. 3.**
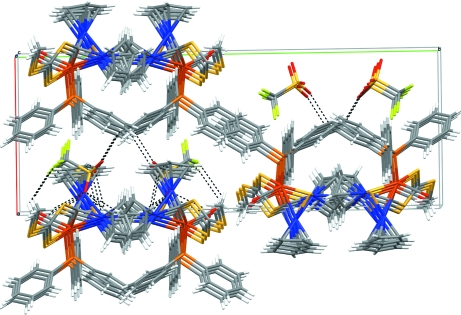
Crystal packing of (I) viewed down the c axis.

## supplementary crystallographic information

### Comment

The title compound (I) was first reported and characterized by us (Manning *et al.*, 2003), as part of a study into the reaction of carbon disulfide with the µ_3_-CS cluster [{Co(η^5^-C_5_H_5_)}_2_{Fe(CO)(PPh_3_)}(µ_3_-S)(µ_3_-CS)]. The product from this reaction, [{Co(η^5^-C_5_H_5_)}_2_{Fe(CO)(PPh_3_)}(µ_3_-S)(µ_3_-C_2_S_3_)], reacted with alkylating agents Me*X* to give [{Co(η^5^-C_5_H_5_)}_2_{Fe(CO)(PPh_3_)}(µ_3_-S)(µ_3_-C_2_S_3_Me)]\ ^+^[*X*]^-^ salts. The compound with [*X*]^-^ = I^-^ was characterized crystallographically in the initial report. Since then crystals of the compound (I) where [*X*]^-^ is trifluoromethanesulfonate have come to hand allowing us to determine the effect of the counter-anion on the unusual structure of the cation.

The asymmetric unit of (I), C_32_H_28_OPS_4_FeCo_2_^+^, CO_3_F_3_S^-^, consists of a bicapped iron-dicobalt cluster cation and a trifluoromethanesulfonate anion (Fig. 1). The structure of the cation in (I) is very similar to that of the cation in the previously reported iodide salt [{Co(η^5^-C_5_H_5_)}_2_{Fe(CO)(PPh_3_)}(µ_3_-S)(µ_3_-C_2_S_3_Me)][I], Manning *et al.* (2003). The bond lengths and angles in the cations, (Table 1) are comparable in both structures. They also confirm our suggestion that the bonding within the Fe—S—C(SMe)-S—C metallocycle is delocalized. Bond distances and angles in the anion are also normal (Allen *et al.*, 1987).

In the crystal structure the cation is linked to the anion in the asymmetric unit by a number of weak non-classical C—H···O and C—H···F hydrogen bonds and weak S···O and F···O interactions. A feature of the packing is an intermolecular S···π(Cp) interaction involving the capping S1 atom and the C21···C25 cyclopentadiene ring of an adjacent molecule (Fig. 2), with an S···*Cg*^i^ distance of 3.385Å and a mean S1···*Cg*···Cn angle of 89.9° (*Cg* is the centroid of the C21···C25 cyclopentadiene ring and n = 21···25; symmetry code i = *x*, 3/2 - *y*, 1/2 + *z*). Such interactions between S atoms and benzene rings are common, 1781 examples with S···*Cg* distances in the range 3.0 ··· 3.7 Å (mean 3.54 Å) and S1···*Cg*···Cn angles in the range 60···120° (mean 90.0°) in the Cambridge database Ver 5.29 to January 2008 (Allen *et al.*, 2004). They are also important in determining protein folding interactions in biochemistry (Ringer *et al.*, 2007). In contrast however, the database reveals only 194 similar interactions involving five-membered aromatic rings with the same distance and angle limitations (mean S···*Cg* distance 3.62 Å, S1···*Cg*···Cn angle 89.9), many of which involve cyclopentadiene rings in transition metal organometallic complexes.

The structure is further stabilized by additional intermolecular C—H···O, C—H···F and O···O contacts which generate an extended network (Table 2). Pairs of cluster cations, interleave with trifluoromethylsulphonate cations to form interlinked columns down the *c* axis (Fig. 3).

For related sulfur and carbon capped triangular FeCo_2_ structures see Manning, O'Dwyer *et al.*, (1995, 1998, 1999); Manning, Palmer *et al.*, (1998).

### Experimental

The title compound was prepared from the room temperature reaction of methyl trifluoromethanesulfonate with [{Co(η^5^-C_5_H_5_)}_2_{Fe(CO)(PPh_3_)}(µ_3_-S)(µ_3_-C_2_S_3_)], Manning *et al.* (2003), with X-ray quality crystals grown from dichloromethane layered with methanol.

### Refinement

The crystals were small and weakly diffracting and little useable data were obtained beyond θ = 24°. All H-atoms bound to carbon were refined using a riding model with d(C—H) = 0.95 Å, *U*_iso_=1.2*U*_eq_ (C) for aromatic and 0.98 Å, *U*_iso_ = 1.5*U*_eq_ (C) for CH_3_ H atoms.

### Crystal data

**Table:**

[FeCo_2_(C_5_H_5_)_2_(C_3_H_3_S_3_)S(C_18_H_15_P)(CO)]CF_3_SO_3_	*F*_000_ = 1840
*M**_r_* = 910.53	*D*_x_ = 1.750 Mg m^−^^3^
Monoclinic, *P*2_1_/*c*	Mo *K*α radiation λ = 0.71073 Å
Hall symbol: -P 2ybc	Cell parameters from 5433 reflections
*a* = 11.0403 (6) Å	θ = 2.3–23.6º
*b* = 29.2183 (14) Å	µ = 1.77 mm^−^^1^
*c* = 10.9040 (5) Å	*T* = 91 (2) K
β = 100.664 (3)º	Irregular fragment, black
*V* = 3456.7 (3) Å^3^	0.18 × 0.06 × 0.06 mm
*Z* = 4	

### Data collection

**Table:**

Bruker APEXII CCD area-detector diffractometer	5502 independent reflections
Radiation source: fine-focus sealed tube	4297 reflections with *I* > 2σ(*I*)
Monochromator: graphite	*R*_int_ = 0.081
*T* = 91(2) K	θ_max_ = 24.2º
ω scans	θ_min_ = 2.0º
Absorption correction: multi-scan(SADABS; Bruker, 2006)	*h* = −12→12
*T*_min_ = 0.717, *T*_max_ = 0.899	*k* = −33→33
36119 measured reflections	*l* = −12→12

### Refinement

**Table:**

Refinement on *F*^2^	Secondary atom site location: difference Fourier map
Least-squares matrix: full	Hydrogen site location: inferred from neighbouring sites
*R*[*F*^2^ > 2σ(*F*^2^)] = 0.031	H-atom parameters constrained
*wR*(*F*^2^) = 0.067	*w* = 1/[σ^2^(*F*_o_^2^) + (0.0227*P*)^2^ + 1.9058*P*] where *P* = (*F*_o_^2^ + 2*F*_c_^2^)/3
*S* = 1.03	(Δ/σ)_max_ = 0.002
5502 reflections	Δρ_max_ = 0.38 e Å^−^^3^
443 parameters	Δρ_min_ = −0.35 e Å^−^^3^
Primary atom site location: structure-invariant direct methods	Extinction correction: none

### Special details

**Table:**

Geometry. All e.s.d.'s (except the e.s.d. in the dihedral angle between two l.s. planes) are estimated using the full covariance matrix. The cell e.s.d.'s are taken into account individually in the estimation of e.s.d.'s in distances, angles and torsion angles; correlations between e.s.d.'s in cell parameters are only used when they are defined by crystal symmetry. An approximate (isotropic) treatment of cell e.s.d.'s is used for estimating e.s.d.'s involving l.s. planes.
Refinement. Refinement of *F*^2^ against ALL reflections. The weighted *R*-factor *wR* and goodness of fit *S* are based on *F*^2^, conventional *R*-factors *R* are based on *F*, with *F* set to zero for negative *F*^2^. The threshold expression of *F*^2^ > σ(*F*^2^) is used only for calculating *R*-factors(gt) *etc*. and is not relevant to the choice of reflections for refinement. *R*-factors based on *F*^2^ are statistically about twice as large as those based on *F*, and *R*- factors based on ALL data will be even larger.

### Fractional atomic coordinates and isotropic or equivalent isotropic displacement parameters (Å^2^)

**Table:**

	*x*	*y*	*z*	*U*_iso_*/*U*_eq_	
S1	0.43507 (7)	0.68025 (3)	0.84869 (8)	0.01321 (19)	
Fe1	0.38034 (4)	0.614761 (15)	0.75881 (4)	0.01093 (12)	
Co1	0.59348 (4)	0.647577 (15)	0.79713 (4)	0.01244 (12)	
Co2	0.43989 (4)	0.690431 (15)	0.65645 (4)	0.01123 (11)	
C1	0.4900 (3)	0.62912 (11)	0.6502 (3)	0.0119 (7)	
S2	0.52197 (7)	0.59798 (3)	0.52361 (8)	0.01445 (19)	
C2	0.3965 (3)	0.56308 (11)	0.5050 (3)	0.0126 (7)	
S3	0.36348 (8)	0.52579 (3)	0.38030 (8)	0.0195 (2)	
C3	0.4831 (3)	0.53630 (12)	0.2912 (3)	0.0219 (8)	
H3A	0.4877	0.5692	0.2747	0.033*	
H3B	0.4642	0.5197	0.2119	0.033*	
H3C	0.5624	0.5258	0.3386	0.033*	
S4	0.29960 (7)	0.56474 (3)	0.60791 (8)	0.0158 (2)	
C4	0.4330 (3)	0.57269 (12)	0.8704 (3)	0.0169 (8)	
O4	0.4722 (2)	0.54557 (8)	0.9446 (2)	0.0268 (6)	
C11	0.7657 (3)	0.65135 (13)	0.7521 (3)	0.0218 (9)	
H11	0.7822	0.6573	0.6712	0.026*	
C12	0.7543 (3)	0.68458 (12)	0.8438 (3)	0.0232 (9)	
H12	0.7608	0.7168	0.8350	0.028*	
C13	0.7314 (3)	0.66107 (13)	0.9513 (3)	0.0242 (9)	
H13	0.7209	0.6748	1.0276	0.029*	
C14	0.7270 (3)	0.61407 (13)	0.9253 (3)	0.0249 (9)	
H14	0.7124	0.5905	0.9807	0.030*	
C15	0.7481 (3)	0.60788 (13)	0.8022 (3)	0.0249 (9)	
H15	0.7501	0.5794	0.7606	0.030*	
C21	0.4574 (3)	0.71066 (11)	0.4791 (3)	0.0171 (8)	
H21	0.4880	0.6920	0.4202	0.021*	
C22	0.3330 (3)	0.71431 (11)	0.4931 (3)	0.0166 (8)	
H22	0.2653	0.6988	0.4441	0.020*	
C23	0.3258 (3)	0.74476 (11)	0.5918 (3)	0.0173 (8)	
H23	0.2534	0.7529	0.6224	0.021*	
C24	0.4474 (3)	0.76111 (11)	0.6372 (3)	0.0187 (8)	
H24	0.4703	0.7827	0.7026	0.022*	
C25	0.5283 (3)	0.73976 (11)	0.5685 (3)	0.0192 (8)	
H25	0.6149	0.7442	0.5802	0.023*	
P1	0.19319 (7)	0.62119 (3)	0.81383 (8)	0.01130 (19)	
C31	0.1058 (3)	0.66800 (11)	0.7289 (3)	0.0118 (7)	
C32	0.0804 (3)	0.66594 (11)	0.5986 (3)	0.0150 (8)	
H32	0.1091	0.6407	0.5572	0.018*	
C33	0.0139 (3)	0.70032 (12)	0.5291 (3)	0.0185 (8)	
H33	−0.0051	0.6980	0.4408	0.022*	
C34	−0.0247 (3)	0.73794 (12)	0.5880 (3)	0.0205 (8)	
H34	−0.0711	0.7614	0.5405	0.025*	
C35	0.0044 (3)	0.74128 (11)	0.7162 (3)	0.0192 (8)	
H35	−0.0199	0.7676	0.7567	0.023*	
C36	0.0686 (3)	0.70657 (11)	0.7865 (3)	0.0159 (8)	
H36	0.0873	0.7092	0.8749	0.019*	
C41	0.1856 (3)	0.63076 (11)	0.9778 (3)	0.0130 (7)	
C42	0.0678 (3)	0.63315 (11)	1.0099 (3)	0.0138 (7)	
H42	−0.0036	0.6317	0.9463	0.017*	
C43	0.0553 (3)	0.63756 (11)	1.1325 (3)	0.0170 (8)	
H43	−0.0244	0.6399	1.1531	0.020*	
C44	0.1591 (3)	0.63864 (11)	1.2259 (3)	0.0157 (8)	
H44	0.1502	0.6407	1.3107	0.019*	
C45	0.2758 (3)	0.63666 (11)	1.1963 (3)	0.0153 (8)	
H45	0.3467	0.6378	1.2606	0.018*	
C46	0.2888 (3)	0.63291 (11)	1.0721 (3)	0.0131 (7)	
H46	0.3688	0.6318	1.0518	0.016*	
C51	0.0968 (3)	0.56975 (11)	0.7843 (3)	0.0135 (7)	
C52	0.0065 (3)	0.56312 (12)	0.6782 (3)	0.0196 (8)	
H52	−0.0116	0.5869	0.6183	0.024*	
C53	−0.0570 (3)	0.52218 (12)	0.6594 (3)	0.0241 (9)	
H53	−0.1173	0.5178	0.5860	0.029*	
C54	−0.0332 (3)	0.48759 (12)	0.7471 (4)	0.0254 (9)	
H54	−0.0778	0.4597	0.7343	0.030*	
C55	0.0555 (3)	0.49360 (12)	0.8532 (3)	0.0239 (9)	
H55	0.0713	0.4701	0.9142	0.029*	
C56	0.1210 (3)	0.53417 (11)	0.8701 (3)	0.0189 (8)	
H56	0.1839	0.5378	0.9417	0.023*	
S5	0.71566 (8)	0.65888 (3)	0.32134 (8)	0.0176 (2)	
O1	0.7829 (2)	0.68745 (8)	0.2496 (2)	0.0249 (6)	
O2	0.59310 (19)	0.64632 (8)	0.2590 (2)	0.0201 (6)	
O3	0.7252 (2)	0.67193 (9)	0.4504 (2)	0.0260 (6)	
C60	0.7989 (3)	0.60449 (13)	0.3295 (3)	0.0221 (8)	
F1	0.78346 (19)	0.58458 (7)	0.21758 (19)	0.0351 (6)	
F2	0.92021 (16)	0.61032 (7)	0.36923 (18)	0.0264 (5)	
F3	0.76096 (18)	0.57506 (7)	0.4081 (2)	0.0321 (5)	

### Atomic displacement parameters (Å^2^)

**Table:**

	*U*^11^	*U*^22^	*U*^33^	*U*^12^	*U*^13^	*U*^23^
S1	0.0146 (4)	0.0129 (5)	0.0127 (4)	−0.0008 (3)	0.0039 (3)	−0.0018 (3)
Fe1	0.0111 (3)	0.0106 (3)	0.0116 (3)	−0.0004 (2)	0.00340 (19)	0.0001 (2)
Co1	0.0102 (2)	0.0157 (3)	0.0112 (2)	−0.00022 (19)	0.00134 (18)	−0.00025 (19)
Co2	0.0121 (2)	0.0103 (2)	0.0113 (2)	−0.00045 (19)	0.00221 (18)	0.00090 (19)
C1	0.0094 (17)	0.0112 (18)	0.0156 (18)	0.0009 (13)	0.0038 (14)	0.0007 (14)
S2	0.0156 (4)	0.0144 (5)	0.0144 (4)	−0.0022 (4)	0.0057 (4)	−0.0032 (3)
C2	0.0123 (17)	0.0109 (18)	0.0148 (18)	0.0048 (14)	0.0035 (14)	0.0019 (14)
S3	0.0193 (5)	0.0198 (5)	0.0198 (5)	−0.0038 (4)	0.0044 (4)	−0.0074 (4)
C3	0.024 (2)	0.025 (2)	0.017 (2)	0.0017 (16)	0.0063 (16)	−0.0051 (16)
S4	0.0140 (4)	0.0157 (5)	0.0188 (5)	−0.0029 (4)	0.0055 (4)	−0.0033 (4)
C4	0.0149 (19)	0.019 (2)	0.019 (2)	0.0005 (15)	0.0081 (15)	−0.0054 (17)
O4	0.0319 (15)	0.0215 (15)	0.0268 (15)	0.0092 (12)	0.0052 (12)	0.0096 (12)
C11	0.0101 (18)	0.043 (3)	0.0125 (19)	−0.0021 (16)	0.0019 (14)	0.0019 (17)
C12	0.0089 (18)	0.024 (2)	0.034 (2)	−0.0051 (15)	−0.0029 (16)	0.0023 (18)
C13	0.0138 (19)	0.044 (3)	0.0131 (19)	0.0004 (17)	−0.0028 (15)	−0.0050 (17)
C14	0.0151 (19)	0.033 (2)	0.023 (2)	0.0009 (17)	−0.0054 (16)	0.0120 (17)
C15	0.0099 (18)	0.033 (2)	0.030 (2)	0.0071 (16)	−0.0024 (16)	−0.0062 (18)
C21	0.024 (2)	0.0151 (19)	0.0141 (19)	0.0052 (15)	0.0093 (15)	0.0054 (15)
C22	0.0197 (19)	0.0156 (19)	0.0129 (18)	0.0019 (15)	−0.0014 (15)	0.0047 (15)
C23	0.0200 (19)	0.0093 (18)	0.023 (2)	0.0055 (15)	0.0046 (15)	0.0030 (15)
C24	0.026 (2)	0.0075 (18)	0.020 (2)	0.0002 (15)	−0.0021 (16)	0.0012 (14)
C25	0.0144 (18)	0.018 (2)	0.025 (2)	−0.0035 (15)	0.0024 (15)	0.0113 (16)
P1	0.0109 (4)	0.0114 (5)	0.0117 (5)	−0.0007 (4)	0.0025 (3)	−0.0005 (4)
C31	0.0084 (17)	0.0115 (18)	0.0165 (19)	−0.0022 (14)	0.0050 (14)	0.0015 (14)
C32	0.0137 (18)	0.0134 (18)	0.0170 (19)	−0.0028 (14)	0.0005 (15)	−0.0010 (15)
C33	0.0173 (19)	0.023 (2)	0.0133 (18)	−0.0009 (16)	−0.0017 (15)	0.0038 (15)
C34	0.0126 (18)	0.024 (2)	0.024 (2)	0.0012 (15)	−0.0001 (15)	0.0086 (16)
C35	0.0207 (19)	0.0122 (19)	0.026 (2)	0.0035 (15)	0.0072 (16)	−0.0013 (16)
C36	0.0184 (19)	0.0155 (19)	0.0151 (18)	−0.0010 (15)	0.0066 (15)	−0.0001 (15)
C41	0.0133 (18)	0.0101 (18)	0.0168 (18)	−0.0008 (14)	0.0057 (14)	0.0011 (14)
C42	0.0125 (18)	0.0143 (19)	0.0138 (18)	−0.0005 (14)	0.0004 (14)	−0.0007 (14)
C43	0.0180 (19)	0.0154 (19)	0.021 (2)	−0.0017 (15)	0.0115 (16)	0.0017 (15)
C44	0.024 (2)	0.0146 (19)	0.0096 (17)	−0.0041 (15)	0.0059 (15)	−0.0016 (14)
C45	0.0166 (19)	0.0146 (19)	0.0132 (18)	−0.0028 (15)	−0.0012 (15)	0.0022 (14)
C46	0.0118 (17)	0.0112 (18)	0.0177 (19)	−0.0010 (14)	0.0059 (14)	−0.0019 (14)
C51	0.0084 (17)	0.0157 (19)	0.0188 (19)	0.0009 (14)	0.0088 (14)	−0.0008 (15)
C52	0.0192 (19)	0.018 (2)	0.021 (2)	−0.0019 (16)	0.0028 (15)	0.0031 (16)
C53	0.0167 (19)	0.024 (2)	0.030 (2)	−0.0065 (16)	−0.0004 (16)	−0.0021 (18)
C54	0.022 (2)	0.016 (2)	0.040 (2)	−0.0047 (16)	0.0102 (18)	−0.0036 (18)
C55	0.025 (2)	0.017 (2)	0.030 (2)	0.0007 (16)	0.0063 (17)	0.0038 (17)
C56	0.0191 (19)	0.017 (2)	0.021 (2)	−0.0005 (16)	0.0035 (15)	0.0013 (16)
S5	0.0152 (5)	0.0214 (5)	0.0160 (5)	−0.0011 (4)	0.0028 (4)	0.0013 (4)
O1	0.0238 (14)	0.0281 (15)	0.0232 (14)	−0.0080 (11)	0.0052 (11)	0.0041 (11)
O2	0.0150 (13)	0.0223 (14)	0.0216 (14)	−0.0027 (10)	−0.0005 (10)	0.0039 (11)
O3	0.0248 (14)	0.0368 (16)	0.0171 (14)	0.0012 (12)	0.0054 (11)	−0.0063 (12)
C60	0.015 (2)	0.034 (2)	0.018 (2)	0.0016 (17)	0.0050 (16)	0.0002 (17)
F1	0.0359 (13)	0.0377 (14)	0.0288 (13)	0.0105 (11)	−0.0013 (10)	−0.0149 (11)
F2	0.0148 (11)	0.0381 (13)	0.0267 (12)	0.0016 (9)	0.0046 (9)	−0.0003 (10)
F3	0.0258 (12)	0.0291 (13)	0.0442 (14)	0.0049 (10)	0.0136 (10)	0.0168 (11)

### Geometric parameters (Å, °)

**Table:**

Co2—S1	2.1275 (9)	C22—C23	1.410 (5)
Co1—S1	2.1564 (9)	C22—H22	0.9500
Fe1—S1	2.1836 (9)	C23—C24	1.425 (4)
Fe1—C4	1.751 (4)	C23—H23	0.9500
Fe1—C1	1.891 (3)	C24—C25	1.413 (5)
Fe1—S4	2.2572 (9)	C24—H24	0.9500
Fe1—P1	2.2634 (10)	C25—H25	0.9500
Fe1—Co1	2.5035 (6)	P1—C31	1.824 (3)
Fe1—Co2	2.6149 (6)	P1—C41	1.827 (3)
Co1—C1	1.867 (3)	P1—C51	1.835 (3)
Co1—C11	2.052 (3)	C31—C36	1.389 (4)
Co1—C15	2.056 (3)	C31—C32	1.398 (4)
Co1—C12	2.060 (3)	C32—C33	1.384 (4)
Co1—C14	2.079 (3)	C32—H32	0.9500
Co1—C13	2.086 (3)	C33—C34	1.380 (5)
Co1—Co2	2.4153 (6)	C33—H33	0.9500
Co2—C1	1.880 (3)	C34—C35	1.379 (5)
Co2—C21	2.065 (3)	C34—H34	0.9500
Co2—C22	2.067 (3)	C35—C36	1.385 (4)
Co2—C23	2.068 (3)	C35—H35	0.9500
Co2—C25	2.073 (3)	C36—H36	0.9500
Co2—C24	2.079 (3)	C41—C46	1.388 (4)
C1—S2	1.743 (3)	C41—C42	1.410 (4)
S2—C2	1.702 (3)	C42—C43	1.375 (4)
S2—F3	3.198 (2)	C42—H42	0.9500
S2—O3	3.317 (3)	C43—C44	1.385 (4)
S2—S5	3.7879 (12)	C43—H43	0.9500
S2—C60	4.032 (4)	C44—C45	1.387 (4)
C2—S4	1.688 (3)	C44—H44	0.9500
C2—S3	1.728 (3)	C45—C46	1.392 (4)
S3—C3	1.805 (3)	C45—H45	0.9500
C3—H3A	0.9800	C46—H46	0.9500
C3—H3B	0.9800	C51—C56	1.391 (4)
C3—H3C	0.9800	C51—C52	1.394 (4)
C4—O4	1.158 (4)	C52—C53	1.382 (5)
C4—O4^i^	4.040 (5)	C52—H52	0.9500
O4—O4^i^	2.941 (5)	C53—C54	1.383 (5)
C11—C15	1.410 (5)	C53—H53	0.9500
C11—C12	1.416 (5)	C54—C55	1.382 (5)
C11—H11	0.9500	C54—H54	0.9500
C12—C13	1.421 (5)	C55—C56	1.383 (5)
C12—H12	0.9500	C55—H55	0.9500
C13—C14	1.401 (5)	C56—H56	0.9500
C13—H13	0.9500	S5—O1	1.440 (2)
C14—C15	1.416 (5)	S5—O3	1.443 (2)
C14—H14	0.9500	S5—O2	1.445 (2)
C15—H15	0.9500	S5—C60	1.829 (4)
C21—C22	1.414 (4)	C60—F1	1.334 (4)
C21—C25	1.415 (5)	C60—F3	1.335 (4)
C21—H21	0.9500	C60—F2	1.340 (4)
			
Co2—S1—Co1	68.64 (3)	C13—C12—Co1	70.94 (19)
Co2—S1—Fe1	74.67 (3)	C11—C12—H12	126.2
Co1—S1—Fe1	70.45 (3)	C13—C12—H12	126.2
C4—Fe1—C1	114.96 (14)	Co1—C12—H12	124.9
C4—Fe1—S1	105.94 (11)	C14—C13—C12	108.1 (3)
C1—Fe1—S1	86.02 (10)	C14—C13—Co1	70.05 (19)
C4—Fe1—S4	95.02 (11)	C12—C13—Co1	68.97 (19)
C1—Fe1—S4	84.22 (10)	C14—C13—H13	125.9
S1—Fe1—S4	159.02 (4)	C12—C13—H13	125.9
C4—Fe1—P1	94.37 (11)	Co1—C13—H13	126.6
C1—Fe1—P1	150.38 (10)	C13—C14—C15	108.2 (3)
S1—Fe1—P1	89.92 (3)	C13—C14—Co1	70.6 (2)
S4—Fe1—P1	89.44 (3)	C15—C14—Co1	69.12 (19)
C4—Fe1—Co1	88.38 (11)	C13—C14—H14	125.9
C1—Fe1—Co1	47.82 (9)	C15—C14—H14	125.9
S1—Fe1—Co1	54.27 (3)	Co1—C14—H14	125.9
S4—Fe1—Co1	127.00 (3)	C11—C15—C14	108.1 (3)
P1—Fe1—Co1	143.12 (3)	C11—C15—Co1	69.8 (2)
C4—Fe1—Co2	144.34 (11)	C14—C15—Co1	70.84 (19)
C1—Fe1—Co2	45.92 (9)	C11—C15—H15	126.0
S1—Fe1—Co2	51.69 (3)	C14—C15—H15	126.0
S4—Fe1—Co2	109.44 (3)	Co1—C15—H15	125.0
P1—Fe1—Co2	110.91 (3)	C22—C21—C25	107.7 (3)
Co1—Fe1—Co2	56.263 (16)	C22—C21—Co2	70.06 (18)
C1—Co1—C11	104.89 (14)	C25—C21—Co2	70.28 (19)
C1—Co1—C15	103.48 (14)	C22—C21—H21	126.1
C11—Co1—C15	40.14 (14)	C25—C21—H21	126.1
C1—Co1—C12	136.54 (15)	Co2—C21—H21	125.1
C11—Co1—C12	40.27 (14)	C23—C22—C21	108.9 (3)
C15—Co1—C12	67.43 (14)	C23—C22—Co2	70.08 (18)
C1—Co1—C14	133.31 (14)	C21—C22—Co2	69.91 (18)
C11—Co1—C14	67.24 (14)	C23—C22—H22	125.6
C15—Co1—C14	40.04 (14)	C21—C22—H22	125.6
C12—Co1—C14	67.03 (14)	Co2—C22—H22	126.0
C1—Co1—C13	170.24 (14)	C22—C23—C24	107.1 (3)
C11—Co1—C13	67.19 (14)	C22—C23—Co2	70.05 (18)
C15—Co1—C13	66.83 (14)	C24—C23—Co2	70.33 (18)
C12—Co1—C13	40.08 (14)	C22—C23—H23	126.5
C14—Co1—C13	39.32 (14)	C24—C23—H23	126.5
C1—Co1—S1	87.40 (10)	Co2—C23—H23	124.8
C11—Co1—S1	150.50 (11)	C25—C24—C23	108.4 (3)
C15—Co1—S1	162.20 (11)	C25—C24—Co2	69.87 (19)
C12—Co1—S1	114.00 (11)	C23—C24—Co2	69.47 (18)
C14—Co1—S1	122.79 (11)	C25—C24—H24	125.8
C13—Co1—S1	102.27 (10)	C23—C24—H24	125.8
C1—Co1—Co2	50.10 (10)	Co2—C24—H24	126.4
C11—Co1—Co2	113.13 (10)	C24—C25—C21	107.9 (3)
C15—Co1—Co2	142.31 (11)	C24—C25—Co2	70.35 (19)
C12—Co1—Co2	110.88 (10)	C21—C25—Co2	69.72 (19)
C14—Co1—Co2	176.57 (11)	C24—C25—H25	126.0
C13—Co1—Co2	137.37 (11)	C21—C25—H25	126.0
S1—Co1—Co2	55.12 (3)	Co2—C25—H25	125.5
C1—Co1—Fe1	48.64 (10)	C31—P1—C41	105.44 (15)
C11—Co1—Fe1	149.80 (10)	C31—P1—C51	106.86 (14)
C15—Co1—Fe1	122.58 (11)	C41—P1—C51	99.61 (15)
C12—Co1—Fe1	169.27 (10)	C31—P1—Fe1	110.52 (11)
C14—Co1—Fe1	117.35 (10)	C41—P1—Fe1	118.79 (11)
C13—Co1—Fe1	136.90 (10)	C51—P1—Fe1	114.43 (11)
S1—Co1—Fe1	55.28 (3)	C36—C31—C32	118.3 (3)
Co2—Co1—Fe1	64.199 (18)	C36—C31—P1	123.5 (2)
C1—Co2—C21	99.18 (14)	C32—C31—P1	118.1 (2)
C1—Co2—C22	114.54 (13)	C33—C32—C31	120.8 (3)
C21—Co2—C22	40.03 (12)	C33—C32—H32	119.6
C1—Co2—C23	152.15 (13)	C31—C32—H32	119.6
C21—Co2—C23	67.54 (13)	C34—C33—C32	120.1 (3)
C22—Co2—C23	39.88 (13)	C34—C33—H33	119.9
C1—Co2—C25	118.61 (14)	C32—C33—H33	119.9
C21—Co2—C25	40.00 (13)	C35—C34—C33	119.6 (3)
C22—Co2—C25	67.01 (13)	C35—C34—H34	120.2
C23—Co2—C25	67.53 (13)	C33—C34—H34	120.2
C1—Co2—C24	157.72 (14)	C34—C35—C36	120.6 (3)
C21—Co2—C24	66.98 (14)	C34—C35—H35	119.7
C22—Co2—C24	66.74 (13)	C36—C35—H35	119.7
C23—Co2—C24	40.20 (12)	C35—C36—C31	120.5 (3)
C25—Co2—C24	39.79 (13)	C35—C36—H36	119.7
C1—Co2—S1	87.93 (10)	C31—C36—H36	119.7
C21—Co2—S1	170.52 (10)	C46—C41—C42	118.8 (3)
C22—Co2—S1	141.40 (10)	C46—C41—P1	123.5 (2)
C23—Co2—S1	108.56 (10)	C42—C41—P1	117.5 (2)
C25—Co2—S1	130.76 (10)	C43—C42—C41	120.6 (3)
C24—Co2—S1	104.29 (10)	C43—C42—H42	119.7
C1—Co2—Co1	49.63 (9)	C41—C42—H42	119.7
C21—Co2—Co1	124.51 (9)	C42—C43—C44	119.9 (3)
C22—Co2—Co1	160.72 (10)	C42—C43—H43	120.0
C23—Co2—Co1	157.87 (9)	C44—C43—H43	120.0
C25—Co2—Co1	108.60 (9)	C43—C44—C45	120.4 (3)
C24—Co2—Co1	122.85 (9)	C43—C44—H44	119.8
S1—Co2—Co1	56.25 (3)	C45—C44—H44	119.8
C1—Co2—Fe1	46.27 (10)	C44—C45—C46	119.8 (3)
C21—Co2—Fe1	135.74 (10)	C44—C45—H45	120.1
C22—Co2—Fe1	120.30 (10)	C46—C45—H45	120.1
C23—Co2—Fe1	127.53 (10)	C41—C46—C45	120.4 (3)
C25—Co2—Fe1	164.27 (10)	C41—C46—H46	119.8
C24—Co2—Fe1	154.27 (10)	C45—C46—H46	119.8
S1—Co2—Fe1	53.64 (3)	C56—C51—C52	118.2 (3)
Co1—Co2—Fe1	59.538 (17)	C56—C51—P1	117.4 (2)
S2—C1—Co1	130.47 (17)	C52—C51—P1	124.2 (3)
S2—C1—Co2	129.07 (18)	C53—C52—C51	120.6 (3)
Co1—C1—Co2	80.27 (13)	C53—C52—H52	119.7
S2—C1—Fe1	128.84 (18)	C51—C52—H52	119.7
Co1—C1—Fe1	83.54 (13)	C52—C53—C54	120.2 (3)
Co2—C1—Fe1	87.81 (13)	C52—C53—H53	119.9
C2—S2—C1	97.32 (15)	C54—C53—H53	119.9
C2—S2—F3	122.72 (12)	C55—C54—C53	120.1 (3)
C1—S2—F3	136.38 (11)	C55—C54—H54	120.0
C2—S2—O3	159.01 (12)	C53—C54—H54	120.0
C1—S2—O3	95.49 (11)	C54—C55—C56	119.5 (3)
F3—S2—O3	52.94 (6)	C54—C55—H55	120.3
C2—S2—S5	138.33 (11)	C56—C55—H55	120.3
C1—S2—S5	115.77 (11)	C55—C56—C51	121.4 (3)
C2—S2—C60	130.20 (12)	C55—C56—H56	119.3
C1—S2—C60	132.48 (12)	C51—C56—H56	119.3
S4—C2—S2	120.38 (19)	O1—S5—O3	115.12 (15)
S4—C2—S3	118.30 (18)	O1—S5—O2	115.05 (14)
S2—C2—S3	121.31 (19)	O3—S5—O2	114.79 (14)
C2—S3—C3	104.35 (16)	O1—S5—C60	103.03 (16)
S3—C3—H3A	109.5	O3—S5—C60	103.68 (15)
S3—C3—H3B	109.5	O2—S5—C60	102.73 (15)
H3A—C3—H3B	109.5	O1—S5—S2	172.58 (11)
S3—C3—H3C	109.5	O3—S5—S2	60.23 (10)
H3A—C3—H3C	109.5	O2—S5—S2	64.80 (10)
H3B—C3—H3C	109.5	C60—S5—S2	84.03 (12)
C2—S4—Fe1	106.84 (11)	S5—O3—S2	97.59 (12)
O4—C4—Fe1	177.4 (3)	F1—C60—F3	107.7 (3)
Fe1—C4—O4^i^	165.73 (15)	F1—C60—F2	107.5 (3)
C4—O4—O4^i^	158.3 (3)	F3—C60—F2	106.7 (3)
C15—C11—C12	107.9 (3)	F1—C60—S5	110.9 (2)
C15—C11—Co1	70.08 (19)	F3—C60—S5	112.1 (2)
C12—C11—Co1	70.18 (19)	F2—C60—S5	111.6 (2)
C15—C11—H11	126.0	F1—C60—S2	118.6 (2)
C12—C11—H11	126.0	F2—C60—S2	130.3 (2)
Co1—C11—H11	125.3	S5—C60—S2	69.14 (10)
C11—C12—C13	107.7 (3)	C60—F3—S2	119.84 (19)
C11—C12—Co1	69.55 (19)		

Symmetry codes: (i) −*x*+1, −*y*+1, −*z*+2.

### Hydrogen-bond geometry (Å, °)

**Table:**

*D*—H···*A*	*D*—H	H···*A*	*D*···*A*	*D*—H···*A*
C3—H3A···O2	0.98	2.56	3.477 (4)	156
C3—H3C···F3	0.98	2.62	3.297 (4)	127
C11—H11···O3	0.95	2.41	3.293 (4)	154
C21—H21···O2	0.95	2.64	3.588 (4)	174
C21—H21···O3	0.95	2.64	3.235 (4)	121
C13—H13···O1^ii^	0.95	2.42	3.288 (4)	152
C14—H14···F1^ii^	0.95	2.56	3.248 (4)	129
C35—H35···O1^iii^	0.95	2.53	3.283 (4)	136
C24—H24···O2^iv^	0.95	2.49	3.298 (4)	143

Symmetry codes: (ii) *x*, *y*, *z*+1; (iii) *x*−1, −*y*+3/2, *z*+1/2; (iv) *x*, −*y*+3/2, *z*+1/2.
